# Review of Experience of the Production of Salt Fortified with Iron and Iodine

**DOI:** 10.1093/jn/nxaa279

**Published:** 2021-02-15

**Authors:** Alister Shields, Mohammed Anas Ansari

**Affiliations:** Independent Consultant, Pretoria, South Africa; Independent Consultant, Lucknow, India; Former Salt Commissioner of India, Office of the Salt Commissioner, Department for Promotion of Industry and Internal Trade, Jaipur, India

**Keywords:** food fortification, double fortified salt, iron, iodine, fortificant production, fortification processes, cost of fortification, commercial challenges

## Abstract

The double fortification of salt with iodine and iron has been proposed as a method for the mass prevention of iron deficiency anemia. This article reports on the technical and financial aspects of the production of such double fortified salt (DFS) based on the experiences of current and past producers. It draws contrasts with the established process of fortifying salt solely with iodine particularly examining the cost and complexity of the processes involved. Based on these factors it questions the commercial viability of existing DFS formulations and thus their sustainability as vehicles for the widespread distribution of iron outside a subsidized environment. It makes suggestions for the future development of DFS particularly relating to the development of less expensive iron formulations suitable for use with lower quality salts and identifies key technical and economic areas to be taken into account when considering the production of DFS.

## Introduction

Globally, salt iodization has proved a highly successful tool for the widespread delivery of iodine, an essential nutrient, to the general population and thus has helped to prevent iodine deficiency disorders. Based on this success, the double fortification of salt with iodine and iron to prevent iron deficiency anemia (IDA) was proposed as early as 1969 (1). The challenge of preventing interactions between the iron and iodine meant that significant production of double fortified salt (DFS) only commenced in the early 2000s. In the intervening period, various formulations and processes were trialed in order to produce a stable product (2). Although research involving iron compounds and formulations for use in DFS continues, DFS is now produced in the private sector for distribution in both commercial and social safety net channels. Fortification of any food requires the industry producing that food to have the necessary knowledge and equipment to adequately fortify according to any required standards or specifications. Currently, DFS production is relatively limited, both geographically and in volume. Considering interests from the global public health community in the viability of scaling up production of DFS, there is a need to describe the private sector's past and current experiences of producing and marketing DFS to inform future feasibility.

The objective of this article is to describe the products and processes in current use to produce the iron formulations used in DFS and the blending process to produce DFS. We discuss technical requirements for DFS production, the costs involved, and the challenges that these may pose to salt producers.

As DFS potentially proposes to leverage the coverage of existing salt iodization programs, considering any lessons learned in private sector production of salt iodization is key. Applying this article's findings will allow governments or producers contemplating DFS to understand the requirements for integrating DFS into their national salt supply.

## Methods

A desk review was conducted to determine the current extent of DFS production, iron formulations, and processes in use, and identify producers of DFS (present and past) and iron formulations. We contacted nongovernmental organizations (NGO) currently active in advocating for or implementing DFS programs, such as Nutrition International (NI), formerly the Micronutrient Initiative, and the Global Alliance for Improved Nutrition (GAIN) to gather further information.

Based on the information collected in the desk review, we contacted each of the DFS producers in India to discuss their current production processes and experiences, including the use of various iron formulations for DFS and their market for DFS. There are 24 producers of whom 20 were interviewed in-person using a standard set of questions. Present and past DFS producers in other countries were contacted and key informant interviews were conducted via phone or e-mail when possible. If these producers could not be contacted, then information on their DFS activities was gathered through interviews with other organizations engaged in support of these activities (e.g., NI and GAIN).

## The DFS Production Process

**FIGURE 1:**
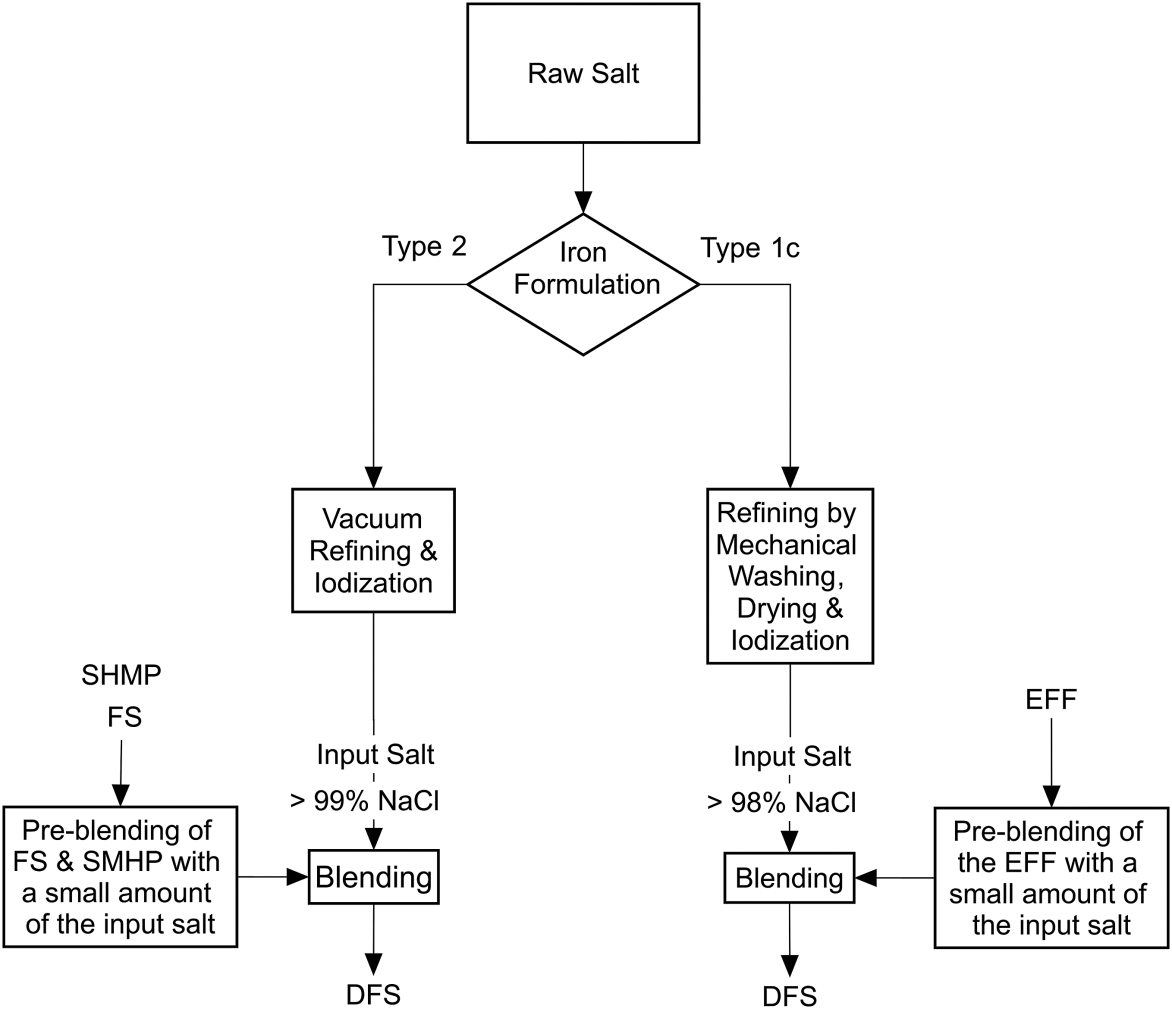
The production process for the 2 most common types of DFS. DFS, double fortified salt; EFF, encapsulated ferrous fumarate; FS, ferrous sulfate; NaCl, sodium chloride; SHMP, sodium hexametaphosphate.

**Figure 1** provides an overview of the DFS production process. Each step in the process is reviewed in detail below.

### The inputs

#### Iron formulations

Initially, the focus of DFS production was on stabilizing the iodine to prevent adverse reactions with the iron. However, with the widespread availability of already iodized salt, the current DFS production practice has shifted to producing an iron formulation stabilized to prevent adverse reactions between iron and iodine. Current DFS production is based on the addition of iron to salt already fortified with iodine. There is a large global market for iodine, in the vicinity of 30,000 metric tons per annum (tpa), of which ∼4% is used for salt iodization (3). This results in a secure supply of iodine, with a wide number of potential suppliers, and ensures competitive pricing. The most widely used form of iodine compound, due to its stability, is potassium iodate. A small number of countries including the USA and Canada also use potassium iodide. Background information on the use of iodine compounds for salt fortification, including the physical properties of various compounds, their use in different countries, fortification concentrations used, and procurement specifications, is summarized on the Iodine Global Network's website (4). In contrast, the production of the iron compounds used in DFS is an ongoing process of evolution, driven by the need to avoid chemical interactions between iron and iodine. There are currently 5 iron formulations, classified as Types 1b, 1c, 2, 3, and 5, in active use (5).

##### Types 1b/1c

There are currently 2 types of encapsulated ferrous fumarate (EFF) in use. Type 1b is produced using a fluidized bed to produce EFF pellets, which are then coated with encapsulating agents. In Type 1c, the EFF is extruded with a binding agent [hydroxy propyl methyl cellulose (HPMC)] and cut into pellets. The pellets are then coated with a color stabilizer [sodium hexametaphosphate (SHMP)] and a color masking agent [titanium dioxide (TiO_2_)] before final encapsulation in a soy stearate coating along with additional TiO_2_.

The size of the pellets for both types is important as it needs to closely match the grain size of the salt that it will be blended with. Homogenous particle sizes are intended to ensure even mixing and avoid separation via settling.

Unlike other iron formulations that have been used in DFS, EFF requires a separate manufacturing process in order to produce the pellets; in current DFS production, the EFF used in Type 1b and Type 1c is externally purchased by DFS producers for blending. A previous iteration of ferrous fumarate (Type 1a) used in DFS production did not use encapsulation and is no longer in use as it caused significant organoleptic changes in the final product. In present DFS programs, Type 1c has largely replaced the use of Type 1b. This is due to the appearance of black spots in DFS produced using Type 1b.

##### Type 2

Ferrous sulfate (FS) was pioneered as an iron compound used in DFS by the National Institute of Nutrition, India (NIN). Type 2 DFS uses ferrous sulfate heptahydrate as an iron source with SHMP added as a color stabilizer. These are added to salt at the rate of 0.5% and 1%, respectively. Both products are widely available in the chemical market.

##### Type 3 (with additional nutrients)

A single producer in India is using a chelated FS to produce a multiple fortified salt with iron, iodine, folic acid, zinc, vitamin A, and vitamin B-12. From 1998 onwards, a fine table salt was produced with the above micronutrients. In 2011, the organization also began fortifying coarser crystal salt, which is used more widely in India omitting the vitamin A (M Vinodkumar, Sundar Serendipity Foundation, personal communication, 2019).

##### Type 5

Micronized ferric pyrophosphate (MFPP) is currently in use in Argentina. The composition of the MFPP formulation used, including whether any additives are used and blending ratio, is not known. The producer has not experienced any difficulties sourcing MFPP but did state that the cost was a significant component of the higher price of the company's DFS product compared with its iodized salt.

### Input salt

An important factor in the successful production of DFS is the quality of the input salt. In many countries nearly all salt used for human consumption is raw solar salt, which is produced by the multistage evaporation of sea water in large outdoor ponds. The salt is scraped from the final stage ponds and stacked for later use. It may then be iodized and sold directly by the producer or sent for further processing, as described below. Apart from the ponds and simple pumps, raw solar salt production requires little infrastructure and in suitable areas is often carried out by multiple producers of varying sizes, with production volumes ranging from 10s to 100,000s of tpa.

The process of iodizing salt is simple, involving the mixing of a solution of iodine, in the form of either potassium iodate or potassium iodide, with the salt. This is usually done by spraying or drop feeding the solution onto the salt at the beginning of a mixing process, which ensures homogeneity of the iodized salt. The salt may also be refined prior to iodization through a process of washing and drying to produce refined salt with an NaCl content of >98%, but this is optional to meet consumer requirements for whiter salt and not required for iodization.

Iodine stability in salt is affected by certain salt qualities, in particular, the moisture content and magnesium (Mg) concentration. To ensure adequate iodine retention, most countries have adopted a minimum standard for iodized salt. These typically require an NaCl content of ≥96% with a moisture content of <4% and <0.5% water-soluble Mg. **Supplementary Table 1** shows a typical standard, the East African regional standard 35:2011, for iodized salt for human consumption.

India is the only country with a standard for DFS, IS 16232:2014, which allows Types 1b/1c (EFF) and 2 (FS) to be used for DFS production. The standard is issued by the Bureau of India Standards (BIS) and is separate from the other fortification standards including those for other food-grade salts, which come from the Food Safety and Standards Authority of India (FSSAI). The BIS standard specifies not only the quality of the final product (DFS) but also the quality of input salt to be used for fortification (see Supplementary Table 1). The quality of input salt required is higher than that specified for iodization and differs depending on whether fortification uses FS or EFF. For fortification with FS an NaCl content of ≥99% is required while fortification with EFF requires ≥98% NaCl. Lower moisture (<1.5%) and Mg contents (<0.1%) are also specified. It is unclear to the authors how the input salt quality requirements were developed and whether lower quality salt may also be adequate in DFS production – outside of India, there is no other country in the world with a national DFS standard for comparison (Ethiopia includes iron in its salt standard, but the concentrations specified are so low that it may be referring to iron contaminant limits rather than the addition of a nutrient for public health benefit) (6). Compared with the salt quality specified in the East African standard, the quality specified for salt to be used to produce DFS in India is much higher. It specifies a higher NaCl concentration and much lower moisture content and magnesium concentration. Even in India, with its well-developed salt industry, only a limited number of salt producers and processors can produce salt that meets the standard set for DFS production.

To meet higher quality salt standards, raw solar salt needs further processing by refining. An initial challenge is to obtain adequate quantities of raw salt of suitable quality for such refining. To ensure the technical and economic feasibility of the refining process, losses (the amount of all material lost during refining) should be in the 8–10% range – which requires salt with an NaCl content of ≥94%. In many countries, much of the salt production is by smaller producers. Although they have the potential to produce salt of this quality, they often lack the technical skills and capacity to carry out the necessary quality assurance and control processes to do so. These small producers are often numerous and spread over distant locations, making external facilitation and monitoring from government bodies difficult. As a result, the losses are much higher. From the authors’ experience these can be as high as 30%. Even where larger producers exist, they often fail to produce raw salt suitable for refining. Samples of raw salt from medium-scale (1000s tpa) producers taken by the author in Mozambique had NaCl contents ranging from 87 to 91%, indicating losses in the 14–21% range. Experience from Universal Salt Iodization (USI) programs suggests that quality improvements among smaller producers are possible, but difficult. For example, in the authors' experience, in Bangladesh, producers were able to increase NaCl concentrations in locally produced raw salt from 78–80% to 97%. However, such improvements require significant commitment from both government and industry to improve production practices and strengthen external support to producers.

To achieve 98% NaCl and reduce moisture levels, raw salt is refined using a process of mechanical washing, centrifuging, drying, grinding, and sieving. Any modern salt refinery capable of carrying out this process should be technically capable of producing salt with ≥98% NaCl content. The vast majority of salt produced in this manner is used in the chemical industry and there are numerous refineries around the world that produce for this market.

Reaching a 99% NaCl content requires vacuum refining of the salt, in which mechanical washing of the salt is replaced by vacuum processing at a high temperature. This is a complex, energy-intensive, and highly industrialized process. Due to the high levels of initial investment and ongoing operating costs, vacuum refining of salt is only done in large-scale operations. There are 2 large producers in India with a combined capacity of >1 million tpa, where a significant proportion of the production is used for table salt. Outside India there are about 15 large manufacturers of vacuum salt and its primary use is in the chemical industry and for pharmaceuticals.

The existing Indian DFS standard is missing an important requirement for salt to be used in DFS: the need for a consistent salt grain size closely matched with that of the fortificant pellets. Currently, iron formulations are dry mixed with salt and thus remain physically separate. Unless the fortificant particles are very small and able to adhere to the salt, as may be the case with MFPP, the size of the fortificant particles must not differ too much from that of the salt. If the size difference is too big then separation may occur during packing, transportation, and storage of the DFS, resulting in a significant lack of homogeneity within the package when it reaches the final consumer.

### Production process for DFS: blending iodized salt with iron

The addition of iron to already iodized salt is a simple process, requiring the dry mixing of the iron formulation with the salt. If producing Type 2 DFS (using FS and SHMP), this is a two-stage blending process starting with the production of a premix of salt with ferrous sulphate and SHMP which is then followed by the premix being blended with salt and dried. If producing Type 1b/1c DFS (using encapsulated ferrous fumarate), the encapsulated ferrous fumarate is first mixed with iodized salt at a ratio of 1:10. The resulting mixture is then added to the rest of the bulk iodized salt and mixed to form the final DFS product, using larger ribbon blenders or screw mixers. The blending process may affect the integrity of the encapsulated formulations via high temperatures of more than 60°C and through abrasion damaging the protective coatings on the premix grains. The equipment must therefore be operated to ensure that the temperature of the salt does not exceed 60°C and must be designed to minimize the shear forces generated during mixing. For small volumes, batch processing using ribbon blenders is often used. Batch processing however is inherently inefficient; continuous processing is preferable for larger volumes.

The FS used in the Type 2 formulation is classified as a skin, eye, and nasal irritant (7). Producers using this formulation have reported health issues with their workers, such as skin rashes and respiratory problems. Skin irritation issues have also been encountered with the handling of ortho-phosphoric acid, which is sometimes used to adjust the salt pH to the range specified in the Indian standard.

Most Indian producers have ceased using the Type 2 formulation, citing several reasons: the high cost of both procuring the components of the formulation and procuring or refining 99% NaCl salt; the impact of even small changes in salt quality on the appearance of the DFS which takes on a yellow color; and the high costs of staff training and quality control/quality assurance measures necessary to produce a consistent product.

Of the 20 producers who responded to the survey, only 3 DFS producers currently use FS, and a fourth will produce DFS with FS only on request. Fifteen producers reported using EFF, with 1 using FS with unspecified chelating additives.

### The product – DFS

After final mixing, DFS is packed for distribution and sale. Most salt standards for human consumption include packaging and labeling requirements. Typical salt packaging ranges from small 250 g to 1 kg retail packs for direct sale to consumers, up to sacks of 50 kg for bulk use. The predominant materials for retail packaging are low-density polyethylene (LDPE) coextruded with linear low-density polyethylene (LLDPE) and often laminated with polyethylene terephthalate. In addition, bulk packaging consisting of woven sacks lined with the same materials is often allowed.

The requirements for packaging DFS to maintain product stability are less clear given the limited production globally. However, there is evidence (8) that the interaction between iron and iodine after blending can cause discoloration of packaging materials. As a result, The India Nutrition Initiative, an NGO specializing in DFS in India, advises that a nonlaminated polymer blend should be used for DFS packaging: LDPE 1005FY Grade 20%, Metelosence FPS117 D 50%, Octane 019010 30%.

Additionally, labeling requirements for DFS will likely differ on a country-by-country basis. At a minimum, traceability data (producer name, batch number etc.) would likely be included but nationally there are often additional requirements of fortified foods. For example in India, fortified products must use a fortification logo and state the nutrients the food is fortified with; products fortified with iron must also state “People with thalassemia may take under medical supervision” (9).

## The Financial Aspects of DFS Production

### Capital investments

Depending on the existing capacity of a salt production and refining industry, the production of DFS may require multiple levels of investment:

#### Investments to produce raw salt of adequate quality for refining

If domestically produced raw salt is of low quality, an initial investment may be required to improve the quality of raw salt produced to a level where it is suitable for refining. The level of investment required for this is highly dependent on the nature of the salt industry in the country concerned and based on factors such as number, size, and geographical dispersal of the raw salt producers, current production practices, and existing capacity of regulatory bodies to ensure raw salt quality.

#### Investments to produce refined salt meeting requirements for DFS

Once salt of a suitable quality for refining is available, the second level of investment that may be required is in salt refining capacity to produce salt of ≥98% NaCl. A recent quote received by a producer in East Africa for a 7200 tpa refining unit (enough salt for a population of ∼1.8 million, allowing for losses) from Spain was ∼560,000 USD excluding transport to the final location, taxes, and insurance (SH Said, Swahili Coast Salt Company, personal communication, 2019). Allowing for additional costs for land, supporting infrastructure such as water and power connections, transport capacity, etc., the total cost of establishing this plant is currently estimated at 820,000 USD. Again, the exact investment for any given plant is difficult to quantify as it will be dependent on multiple location-specific variables, ranging from land costs to taxation levels.

#### Investments to blend the iron formulation with iodized salt to produce DFS

The final capital investment is in additional equipment to add the iron formulation. The estimated cost of blending equipment with a capacity of 24,000 tpa is USD 71,000, excluding transport from South Africa to the final location, taxes, and insurance. Additional costs include commissioning and installation, land, buildings, and supporting infrastructure necessary for the operation of the equipment. This equipment would be suitable for the production of DFS with both EFF (Types 1b/1c) and FS (Type 2) formulations (N Wildman, Davey Engineering, personal communication, 2019).

As discussed earlier, the EFF (Types 1b/1c DFS formulation) requires a separate manufacturing process. The capital costs to establish a plant to carry out this process are not known at this time. DFS producers are not responsible for capital investments required to produce EFF and it is probable that production will remain with a small group of specialized producers unless the demand for EFF increases massively. The other formulations use compounds that are more widely available and that are added to iodized salt without requiring any further processing.

### Operating costs

Operating costs for DFS producers are difficult to determine, as they are commercially sensitive, and producers are often unwilling to share them. Estimates based on the information provided by the producers indicates the 2 most significant costs are the purchase of input salt (23% and 31% for Type 1c and Type 2, respectively) and for the iron formulation (37% and 25% for Type 1c and Type 2, respectively). **Supplementary Table 2** shows the estimated operating costs to produce Type 1c (EFF) and Type 2 (FS) DFS in more detail.

## Global Experiences of the Production of DFS

### Kenya

During the 2000s, the production of Type 1b DFS was planned. Product acceptability trials were successfully completed; based on these trials, the industry was ready to launch a commercial product. In 2002, production equipment of an approximate current value of 50,000 USD (N Wildman, Davey Engineering, personal communication, 2019) was provided to 2 Kenyan producers with funding from UNICEF and NI. The aim was initially to launch a branded, premium DFS product and then try to expand sales into lower-income groups. However, due to concerns over potential adverse effects of fortifying salt with iron in a malaria-endemic country there was significant resistance from the government, including obstructive regulations, and production never began.

### Nigeria

Nigeria imports all its salt, which is then iodized domestically. Initial efforts to introduce DFS, driven in part by UNICEF, were around mandatory fortification of salt with both iodine and iron and this resulted in significant resistance from processors. Despite this resistance, ∼25,000 USD in dosing and mixing equipment was provided by UNICEF to a Lagos-based salt processor, Royal Salt. Plant trials were carried out and a small amount of Type 1b (EFF) DFS was produced for the open market. It was not considered a successful product primarily due to its higher cost and changes in the salt color after production. Royal Salt returned the equipment, which was later relocated to another Nigerian salt manufacturer, Dangote Salt. As late as 2011, the plant remained idle at Dangote's salt-processing facility in Lagos. After the negative experience from Royal Salt, and the higher cost of producing DFS, Dangote Salt declined to produce DFS without financial support from the government and/or donors to launch a new product.

### Bangladesh

In Bangladesh, DFS was produced using the Type 1b (EFF) formulation and sold during 2011–2012 by Confidence Salt. They were supported by NI, who assisted with equipment procurement, support to the local fabrication of equipment, and with promotional materials. The EFF was purchased from ACG Pharma Technologies Private Ltd (formerly Pam-Glatt Pharma Technologies Private Ltd). The DFS was sold through the company's marketing network at a similar price to its iodized salt products as part of the company's corporate social investment. However, after the results of the 2011–2012 National Micronutrient Survey (10) indicated low levels of iron deficiency and IDA in preschool children, school children, and nonpregnant, nonlactating women, doubts were raised over the need for population-wide iron interventions. NI withdrew its support and Confidence Salt ceased production. A new producer has recently entered the scene and is working closely with NI to produce a premium DFS product, this time using the Type 1c (EFF) formulation. The goal is to sell this new product at an 8–10% increment over the price of iodized salt. They will also target donor-funded projects, such as World Food Programme distributions where the salt is purchased at market value and distributed to vulnerable populations (A Mahfuz, NI, Bangladesh, personal communication, 2019).

### Argentina

Timbó Salt in Argentina is the only producer outside of India who currently produces DFS on a commercial basis purely for the retail market. Production began in 2006 after Timbó bought a company producing low-sodium salt, which uses the same blending equipment that is used to produce DFS. Wanting to do some good and with the equipment available, they first experimented with fortifying low-sodium salt with iron before settling on the fortification of iodized salt with MFPP (Type 5) supplied by a German company, Paul Lohmann GmbH KG. After experimenting with the price, their branded DFS product, known as Celusal Plus, is sold at a 40% wholesale premium over iodized salt of a similar grade, which results in a price differential at the retail level of 50–60%. At this price, a small profit is made, limited by the cost of the iron compound. The product represents only ∼0.5% of the company's sales and is accessible to only ∼40% of the Argentine market. Expanding into other areas of the market did not yield worthwhile increases in sales and the producer believes he has found a happy medium, allowing for an acceptable profit (S Guzman, Timbó Salt, personal communication, 2019). The producer reports the consistent appearance of a slight beige color in the final product, which has been described previously and is 1 of the reasons Type 5 DFS is not in wider use (11). Attempts to market the color change as a visible and positive indicator of added iron were restricted by the government, which is focused on reducing salt consumption by the public.

Timbó Salt reported little change in DFS sales over the last 10 y and expects the situation to remain the same for the foreseeable future.

### India

India is by far the largest producer and consumer of EFF used in Types 1b/1c DFS and of DFS itself. Production is regulated by the FSSAI, which registers all DFS and EFF producers. (**Supplementary Table 3** and **Supplementary Table 4**). There are currently 24 DFS producers and 4 producers of EFF, only 1 of which is actively supplying EFF to DFS producers according to survey results. Two other companies (not listed by FFSAI on its website as registered as of 18 August, 2019) are also supplying EFF to DFS producers. The FSSAI also develops standards for fortified foods, including DFS (12).

Of the 4 registered EFF producers only the two discussed below provided any information to the authors. None of the others responded to requests for information nor could any be found during the desk review.

*Wella Neutralogicals* is an operating trademark of JVS, and JVS has worked in partnership with the University of Toronto (UT) and NI to commercialize the production of EFF using the extrusion process developed by UT, which currently licenses the process to JVS free of charge. JVS has invested ∼1.4 million USD in developing EFF production, along with an unknown amount of direct and indirect support from partners.

*Nutracare* is a private company that has independently developed its own production capacity for EFF. Their exact production process has not been made public

Although FSSAI registers EFF producers there is no standard for the EFF formulation itself.

Registration seems to be a requirement for DFS producers to tender for supply to the various social safety net programs in India.

Although the production of the Type 1 (EFF) formulations is subject to registration, the components for the Type 2 are purchased separately by the DFS producer and added directly to the salt. There is therefore no production process for the formulation and the FSSAI does not require registration.

India has a well-developed salt industry with the capacity to produce salt of the required standard for double fortification and to carry out the blending process for double fortification. Official figures indicate that ∼72% of the iodized salt produced in India meets the BIS standard *Refined Iodized Salt, Vacuum Evaporated Iodized Salt and Iodized Salt* and should be suitable for DFS production. However, in the authors’ experience not all of this salt actually meets the standard; and with variations in composition of salt produced (e.g., high sulfate concentrations) in different areas, even salt meeting the standard could have adverse effects on the final product. Salt meeting the standard for DFS production is also not available in all production areas. Where this is the case, in order to increase availability of DFS-quality input salt, investments to improve raw salt quality and/or create refining capacity would be necessary; alternatively, DFS or salt suitable for the production of DFS would have to be transported from other areas. This would have a significant impact on the existing salt producers and incur additional transport, storage, and handling costs.

There is an excess of DFS production capacity. The producers surveyed have an installed capacity of 914,000 tpa compared with actual production of 117,546 metric tons as of the end of November 2018 (the time of research). Of that production, 85% (100,000 metric tons) was by a single producer.

The national demand for DFS is primarily driven by Indian state governments, where DFS is distributed in social safety net programs, either for free or at a subsidized price. Although the extent of its inclusion in various programs depends on the state, social safety net programs that include DFS are the Public Distribution System, Mid-Day-Meal, and Integrated Child Development Services.

Producers complain that these programs do not create an adequate demand for DFS and therefore they are unable to make a return on their investments in DFS production. They also state several reasons for not being able to sell DFS as a commercial product outside of subsidized social safety net programs: the high price of DFS relative to iodized salt, consumers’ lack of knowledge of the product's benefits, and a perception that it causes sensory changes in food cooked with it (discussed below).

All the producers who currently use or have produced Type 2 (FS) DFS in the past reported color changes in the final product. Two producers also raised concerns about a color change occurring with Type 1c (EFF) DFS.

Of the 7 producers actively producing Type 1c (EFF) DFS (**Supplementary Table 5**), all but 2 reported the appearance of black spots in the final product. Of the 2 that did not report black spots, 1 did not respond to the question. The other reported that black spots were no longer seen due to “significant research” but was unwilling, for commercial reasons, to elaborate what this meant.

## Discussion

DFS was conceived as an innovation to address another persistent nutrient deficiency (iron) by leveraging the success of existing USI efforts. Key factors in the success of USI are the low cost of iodine and the simplicity of the production process, which can be applied to salts of various qualities, needs minimal investment, and incurs low operating costs. Thus, the impact on the consumer price of iodizing salt is minimal, ∼2–3%. In addition, consumers do not perceive any changes in the salt after iodization or in food cooked with it. These features have allowed the widespread adoption of mandatory iodization with little impact on the consumer. That said, many countries have yet to achieve USI despite mandatory legislation being in place, primarily due to reluctance on the part of producers.

The production of DFS however, is significantly more complex, with higher input costs both for the salt and the iron compound. As a result, DFS is substantially more expensive to produce than iodized salt and thus a higher ex-factory price. Various grades of iodized salt are produced and consumed in different sectors of the market. Experience in India indicates an ex-factory price difference ranging from ∼70 to 110% for Type 1c DFS (EFF) and ∼50–90% for Type 2 DFS (FS), depending on the grade of iodized salt to which it is being compared (see **Table 1**). The exact impact of this ex-factory price difference on the consumer price is specific to the conditions of the salt market in the target area. Factors such as transport costs, customary wholesale and retail mark-ups, and taxes all vary greatly and need to be assessed on a case by case basis. In making such assessments, the actual usage patterns of the different grades of iodized salt, especially amongst those most affected by IDA, need to be taken into account when making any comparisons.

**FIGURE 2:**
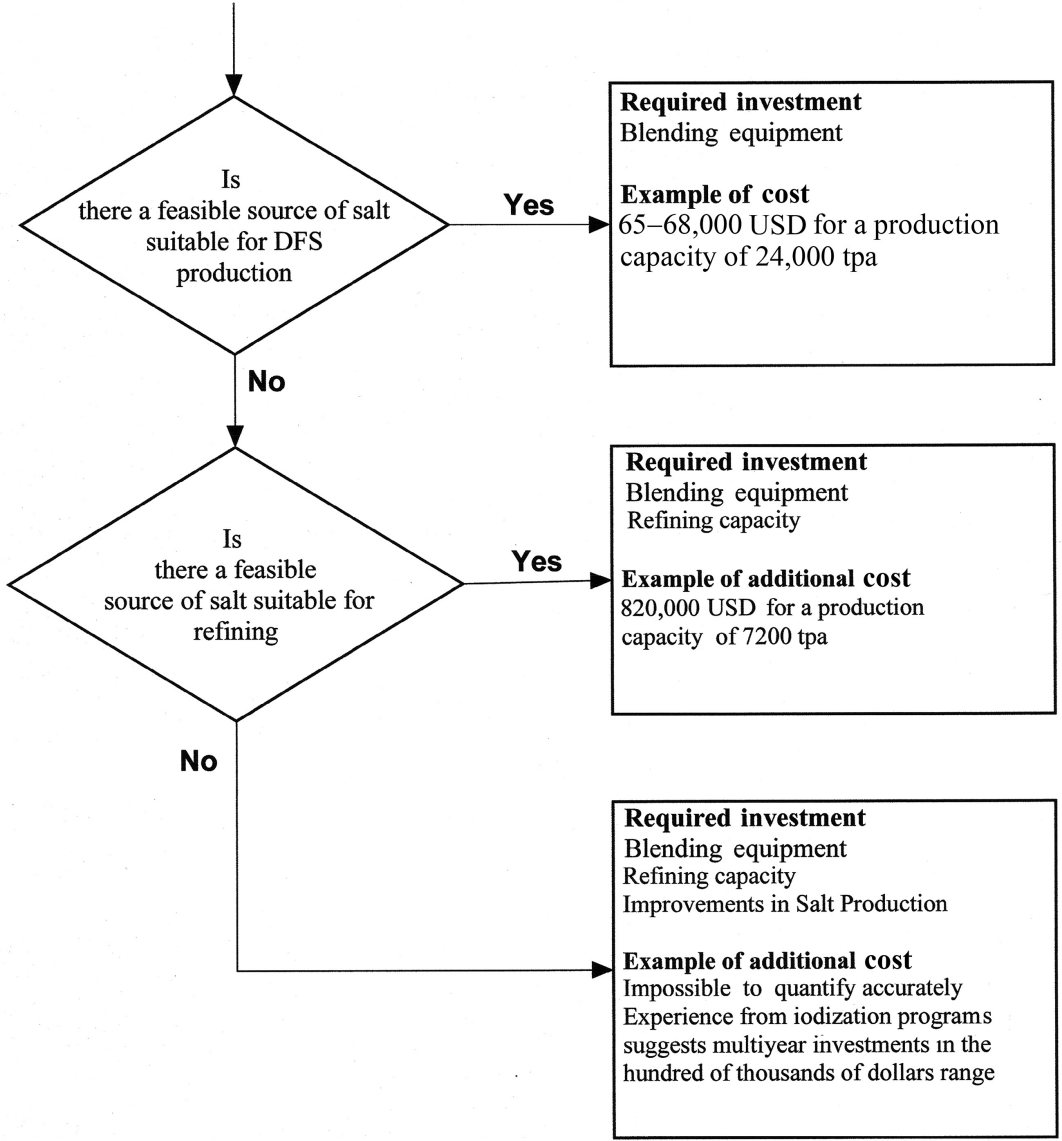
Estimated investments for various DFS production scenarios. NB: Feasible = Sustainable, socially and politically acceptable, and economically viable. Source = local or national production and/or importation.DFS, double fortified salt; tpa, metric tons per annum; USD, United States Dollar.

**TABLE 1:** Comparative costs of DFS produced with Type 1c EFF and FS and various grades of iodized salt in India

		DFS with EFF		DFS with FS	
		Cost = 98.35 *(USD/MT)*		Cost = 89.55 *(USD/MT)*	
Iodized salt grade	Iodized salt cost (*USD/MT)*	Difference (*USD/MT)*	Difference *%*	Difference (*USD/MT)*	Difference *%*
Refined	58.31	40.04	68.7	31.24	53.6
Powdered	48.05	50.30	104.7	41.50	86.4
Crushed	46.65	51.70	110.8	42.90	92.0
Crystal	50.16	48.19	96.1	39.39	78.5

DFS, double fortified salt; EFF, encapsulated ferrous fumarate; FS, ferrous sulfate; MT, metric ton; USD, United States Dollar.

Producers also report color changes in the salt after double fortification (black spots and/or yellowing, depending on the iron compound used) and color and taste changes in food cooked with it. The cause of black spots when using EFF in DFS is unclear. It is possible, for example, that shear forces generated during mixing, particularly at pinch points, are responsible for damaging the coating of some EFF. In this case, further research may be necessary to develop a coating capable of withstanding the shear forces generated during mixing in an industrial setting and/or producers may need to be given guidance in the design and operation of the blending equipment used to produce DFS. Alternatively, the EFF itself may not be of sufficient quality. This is difficult to determine as there is no standard for the production of extruded EFF, in India or globally. Currently, 1 of the quality tests used in the development of EFF is extremely difficult to carry out with samples having to be sent from India to Canada. These factors make it difficult for both the EFF and DFS producers to ensure the quality of the iron formulation. Although EFF producers in India must be registered, given the lack of any standard for the EFF it is difficult to see the impact of this on the quality of EFF produced. Despite lack of registration, 2 unregistered EFF producers supply to DFS producers.

Producers have cited the universal appearance of a yellow color in salt fortified with FS as a significant reason for ceasing production of DFS fortified with FS.

Experience to date in DFS production is limited to producers in a small number of countries that have trialed DFS or produced small amounts but stopped for various reasons (Bangladesh, Kenya, Nigeria), currently produce very small quantities of DFS for the domestic market (Argentina), or rely on government subsidies for maintaining production (India). These experiences suggest that outside of subsidized markets, DFS can only have potential as a premium product that targets higher-income consumers. Such a product is likely to have a limited impact on the prevalence of IDA, which is closely associated with socioeconomic status (13).

## Conclusion

Given the limited production of DFS, and that most of that production has occurred under the unique circumstances prevailing in the Indian market, it is very difficult to draw any definitive conclusions about the feasibility of DFS production. Current experience indicates that at the national level for any given country interested in DFS, there are certain minimum requirements for the production of DFS:

1. A standard for DFS that ensures a high-quality product with a high level of consumer acceptance.
2. A standard for the iron formulations used in DFS, to ensure iodine stability and reduce potential color and taste changes to DFS or in foods cooked with it.
3. An adequate supply of salt of the quality required to allow the production of DFS that meets the standard.
4. An adequate supply of iron formulation meeting the national standard.
5. Salt-processing capacity to mix the salt and iron formulations to produce DFS.
6. Laboratory capacity (or resources to send samples abroad) to test both the iron formulation and DFS for compliance with relevant standards.

**Table 2** raises several questions regarding the feasible incorporation of DFS into existing salt production operations that should be considered by those wishing to engage in the production of DFS for commercial reasons and those supporting its production from a public health perspective, with **Figure 2** providing estimates of the investments required.

**TABLE 2:** Key considerations for salt producers, policymakers, and development partners examining the feasibility of producing DFS

DFS requirement	Salt-producing countries	Countries primarily importing salt
Inputs	Does the salt industry have the capacity to improve the quality of raw salt (if necessary) and develop, own, and operate the refining plant necessary to upgrade the salt to the required standard as well as the blending plant?	Is the imported salt of adequate quality for DFS fortification? If not, can salt of adequate quality be sourced for importation?
	If not, what are the requirements to strengthen industry? For example, by consolidating small producers, improving technical and management capacity, etc., and how much would they cost?	Is there the capacity amongst the importers to carry out the blending of the iron formulation? If not, what would be the cost of installing this capacity?
	Is there a reliable and stable source of the iron formulation and at what price?	Is there a reliable and stable source of the iron formulation and at what price?
Required processing market	What would be the capital cost of establishing the necessary refining and blending plant(s) to produce the required quantity of DFS?	Given that salt is often imported by multiple small companies, who would carry out the fortification (a single service provider, the individual importers etc.?), where would it take place (at a single central location, at multiple location at points of importation etc.?), and who would bear both the capital and operating costs?
	What are the ongoing operating costs?	What are the ongoing operating costs?
	Will the DFS be acceptable to consumers in terms of taste and appearance – of both the DFS and food cooked using it?	Will the DFS be acceptable to consumers in terms of taste and appearance – of both the DFS and food cooked using it?
	Does the proposed production process and DFS meet any existing and proposed standards and regulatory requirements?	Does the proposed production process and DFS meet any existing and proposed standards and regulatory requirements?
	Is there a market for DFS at a price point that will ensure profitability and an adequate return of investment given the costs identified above?	Is there a market for DFS at a price point that will ensure profitability and an adequate return of investment given the costs identified above?
	Are there adequate standards and regulations in place to protect producers from unfair competition from inferior or unfortified products? Is there the capacity and will to enforce them?	
	Where DFS will be used for a subsidized social safety net program, how will sustainability be ensured if funds are no longer available in the future?	Where DFS will be used for a subsidized social safety net program, how will sustainability be ensured if funds are no longer available in the future?

DFS, double fortified salt.

Beyond these questions the biggest obstacle to DFS reaching those most in need is the increased cost of the final product. This is driven primarily by the cost of the formulations used to fortify the salt and the apparent need for high-quality input salt. DFS producers fortifying iodized salt with iron need to obtain a return on the required investments and maintain a reasonable profit, which has not come to pass in existing DFS operations. Many countries will also face challenges with the salt industry's capacity to meet quality requirements at various stages of the DFS production process and the capacity of regulatory bodies to ensure compliance with the standards (if they exist) and regulations necessary for DFS production.

Any future iron formulation research for DFS should aim to address these challenges, by producing an iron formulation meeting the following requirements:

- Can be incorporated in a production process (investments, ongoing costs) yielding DFS that is cost competitive with iodized salt.
- Can be used with salt of a quality that is widely available and in-line with local standards for salt for human consumption.
- Causes no significant changes to the appearance and taste of the final product and in foods cooked with it.
- Can be subjected to simple quality control measures, enabling the elimination of inferior products from the market and building trust between iron formulation manufacturers, salt processors, the government, and consumers.
- Is produced by a process using technology that is amenable to widespread use and using ingredients that are widely available, allowing for local production and avoiding the costs and, in some countries, complexity of importation.

A formulation meeting these requirements would enable the production of DFS at a profit without subsidies, allowing for a return on the investment required to produce DFS for the wider market. It would also result in a product within the reach of lower-income groups that are in most need of dietary interventions to improve iron intake. Without further development, DFS will likely remain a product that will rely on subsidies – either direct or indirect – or will only be available in small niche markets where it will have limited impact on the prevalence of IDA.

## Supplementary Material

nxaa279_Supplementary_Tables

## Notes

AS and MAA were funded by the Iodine Global Network.

Author disclosures: The authors report no conflicts of interest.

The Iodine Global Network played no part in the design, implementation, analysis, and interpretation of the data.

Supplementary Tables 1–5 are available from the “Supplementary data” link in the online posting of the article and from the same link in the online table of contents at https://academic.oup.com/jn/.

Published in a supplement to *The Journal of Nutrition*. The Guest Editor for this supplement was Becky Tsang and she has no conflicts of interest. Publication costs for this supplement were defrayed in part by the payment of page charges by the Iodine Global Network. The opinions expressed in this publication are those of the authors and are not attributable to the sponsors or the publisher, Editor, or Editorial Board of *The Journal of Nutrition*.

The Supplement Coordinator for the supplement publication was Becky Tsang, Iodine Global Network, Seattle, WA, USA.

Abbreviations used: BIS, Bureau of India Standards; DFS, double fortified salt; EFF, encapsulated ferrous fumarate; FS, ferrous sulfate; FSSAI, Food Safety and Standards Authority of India; GAIN, Global Alliance for Improved Nutrition; IDA, iron deficiency anemia; JVS, JVS Foods Private Ltd; LDPE, low-density polyethylene; MFPP, micronized ferric pyrophosphate; Mg, magnesium; NI, Nutrition International; SHMP, sodium hexametaphosphate; TiO_2_, titanium dioxide; tpa, metric tons per annum; UT, University of Toronto; USD, United States Dollar; USI, universal salt iodization.
