# Supplementary material for: Review of Experience of the Production of Salt Fortified with Iron and Iodine
Source: J Nutr. 2021 Feb 15;151(Suppl 1):29S–37S. doi: 10.1093/jn/nxaa279 (PMC7882366; doi:10.1093/jn/nxaa279)
Supplement: nxaa279_Supplementary_Tables [file nxaa279_supplementary_tables.pdf]

Review of Experience of the Production of Salt Fortified with Iron and Iodine

Alister Shields

Online Supplementary Material

Supplementary Table 1: Comparison of Key Quality Parameters for Salt for Iodization vs Salt for Double Fortification

Characteristics	IS 16232:2014 Indian Standard For DFS<br>**INPUT SALT FOR THE PRODUCTION OF DFS**		East African Standard 35:2011 for Iodized Salt<br>**FINAL PRODUCT**			IS 16232:2014 Indian Standard For DFS<br>**FINAL PRODUCT**	
	FS	EFF	Coarse salt	Crushed salt	Table salt	Fortified with EFF	Fortified with FS
Chloride content as (NaCl), % on dry matter basis, min	≥99	≥98	≥96.0	≥96.0	≥97.0	≥97	
Moisture content, drying at 105^0C, %, m/m, max.	≤1.5	≤1.5	≤4	≤4	≤3	≤1.5	
Matter insoluble in water, %, on dry matter basis, max	≤1.0	≤1.0	≤1	≤1	≤0.2	≤1.0	
Magnesium (Mg) water-soluble, % on dry matter basis, max	≤0.1	≤0.1	≤0.5	≤0.5	≤0.1	≤0.1	
Sulphate (as SO4), % on dry matter basis, max	≤1.1	≤1.1	≤0.5	≤0.5	≤0.50	≤1.1	
Acid insoluble matter % m/m, Max	≤0.3	≤0.3	-	-	≤0.2	-	
pH of solution, 20g in 100ml distilled water	3.5 – 5.5	3.5 – 7.5	7.0 - 8.0	7.0 - 8.0	7.0 - 8.0	3.5 – 7.5	3.5 – 5.5

Abbreviations: DFS, double fortified salt; EFF, encapsulated ferrous fumarate: FS, ferrous sulphate; m/m, mass fraction; NaCl, sodium chloride

Supplementary Table 2: Breakdown of Estimated Production Costs for DFS in India

	EFF		FS	
	Cost *(USD/MT)*	% of total cost	Cost *(USD/MT)*	% of total cost
Input Salt	22.48	23%	28.00	31%
Iodization	1.41	1%	1.41	2%
Fortification with Iron Formulation	36.53	37%	22.20	25%
Packaging 1kg Individual	16.16	16%	16.16	18%
Packaging Bulk	5.62	6%	5.62	6%
Processing, Finance, Marketing and Administration	16.16	16%	16.16	18%
Total Cost	98.35		89.55	

Abbreviations: DFS, double fortified salt; EFF, encapsulated ferrous fumarate; FS, ferrous sulphate, MT, metric ton; USD United States Dollar

Supplementary Table 3 Encapsulated Ferrous Fumarate Producers

	Responded to Survey	FSSAI Registered[1]	Production Capacity (tpa)	Actual Production 2017-18	Supplying DFS Producers	No. Of DFS Producers Supplied	EFF Type produced
Wella Neutralogicals	Yes	Yes	1,500	Unknown	Yes	4	Type 1c
Nutracare	Yes	No	1,200	70	Yes	2	Unknown
Salvi Chemical Industries	No	No	Unknown	Unknown	Yes	1	Unknown
Hexagon Nutrition	No	Yes	Unknown	Unknown	No	0	Unknown
Pristine Organics	No	Yes	Unknown	Unknown	No	0	Unknown
Vantage-Nutrition	No	Yes	Unknown	Unknown	No	0	Type 1b

[1]As of September 2019

Abbreviations: DFS, Double Fortified Salt; FSSAI, Food Safety and Standards Authority of India; Tpa, metric tons per annum

Supplementary Table 4: DFS Production by Producer (metric tons per year), India 2016 – 2018[1]

	Production			
DFS Producer	*Quoted Capacity*	*Actual 2016-17*	*Actual 2017-18*	*2018-2019* as at Nov 2018
Ankur	100,000	Unknown	Unknown	100,000
Bajaj	60,000	15,500	15,000	Upgrading
Balaji	60,000	10,000	0	0
Chirai	43,000	5,364	2825	3,846
Chougle	80,000	6000	0	1,000
Goyal[2]	50,000	-	-	0
Indo-Brine	45,000	10,000	32,000	0
Jagannath	64,800	3,187	77.5	505.6
Maheshwari[3]				
Prince International	24,000	-	-	5,000
Sahayamatha Salterns	24,000	-	-	0
Shree Chemfood Pvt Ltd	79,000	16,000	1,700	1,500
Shreeram Chemfood Pvt. Ltd	60,000	-	1,061	434
Sunder Salt[4]				
Super Salt	Unknown	-	26,500	-
Tamil Nadu Salt Corp	10,000	4,796	2,860	5,207
Tata Chemicals[5]				
Terapanth Foods	60,000	624	0	0
Vibrant Salt	120,000	100	210	53
Western	34,200	210	0	0

[1] An additional four licensed producers did not respond to the survey and are not included in the above table
[2] Total production 2016-18 production of 9600t
[3] Currently in setup up phase and not producing
[4] Produces multiple-fortified salt - volumes not supplied
[5] Production outsourced to Shreeram Chemfood above

Abbreviations: DFS, Double Fortified Salt

Supplementary Table 5: Appearance of Black Spots in DFS Amongst Currently Active Producers Using EFF

	**Iron compound in use**		**Appearance of Black Spots in DFS**	
**DFS Producer**	*EFF*	*FS*	*Reported?*	*EFF Source*
Ankur	Yes	On Request	Previously	Nutra Care
Bajaj	Yes	Discontinued	Yes	Wella
Chirai	Yes	No	Yes	Nutra Care
Chougle	Yes	Unknown	No response	Unknown
Jagannath	Yes	Discontinued	Yes	Wella
Shree Chemfood Pvt Ltd	Yes	Yes	Yes	Wella
Shreeram Chemfood Pvt. Ltd	Yes	Discontinued	Yes	Unknown
Vibrant Salt	Yes	No	Yes	Salvi

Abbreviations: DFS, Double Fortified Salt; EFF, encapsulated ferrous fumarate; FS, ferrous fumarate
